# Titanium Dioxide-Based Nanocomposites for Enhanced Gas-Phase Photodehydrogenation

**DOI:** 10.3390/ma12193093

**Published:** 2019-09-23

**Authors:** Danny Zanardo, Elena Ghedini, Federica Menegazzo, Elti Cattaruzza, Maela Manzoli, Giuseppe Cruciani, Michela Signoretto

**Affiliations:** 1 CatMat Lab, Department of Molecular Sciences and Nanosystems, Ca’ Foscari University Venice and Consortium INSTM, RU of Venice, Via Torino 155, 30172 Venezia, Italy; danny.zanardo@unive.it (D.Z.); gelena@unive.it (E.G.); fmenegaz@unive.it (F.M.); 2 Department of Molecular Sciences and Nanosystems, Ca’ Foscari University Venice, Via Torino 155, 30172 Venezia, Italy; cattaruz@unive.it; 3 Department of Drug Science and Technology, University of Turin, 10125, Via P. Giuria 9, 10125 Turin, Italy; maela.manzoli@unito.it; 4 Department of Physics and Earth Sciences, University of Ferrara, Via G. Saragat 1, I-44122 Ferrara, Italy; giuseppe.cruciani@unife.it

**Keywords:** photodehydrogenation, titanium dioxide, copper oxide, ethanol

## Abstract

Light-driven processes can be regarded as a promising technology for chemical production within the bio-refinery concept, due to the very mild operative conditions and high selectivity of some reactions. In this work, we report copper oxide (CuO)-titanium dioxide (TiO_2_) nanocomposites to be efficient and selective photocatalysts for ethanol photodehydrogenation under gas phase conditions, affording 12-fold activity improvement compared to bare TiO_2_. In particular, the insertion method of the CuO co-catalyst in different TiO_2_ materials and its effects on the photocatalytic activity were studied. The most active CuO co-catalyst was observed to be highly dispersed on titania surface, and highly reducible. Moreover, such high dispersion was observed to passivate some surface sites where ethanol is strongly adsorbed, thus improving the activity. This kind of material can be obtained by the proper selection of loading technique for both co-catalysts, allowing a higher coverage of photocatalyst surface (complex-precipitation in the present work), and the choice of titania material itself. Loading copper on a high surface area titania was observed to afford a limited ethanol conversion, due to its intrinsically higher reactivity affording to a strong interaction with the co-catalyst.

## 1. Introduction

Nowadays, the requirement of an alternative, non-fossil-based, energy economy is growing in importance, because the widespread uses of these energy sources is known to have a remarkable negative effect on Earth’s climate [1]. Photocatalysis has been claimed to be a promising technology for the direct conversion of sunlight, the most abundant renewable energy source on the Earth [2], into so-called solar fuels [3]. In recent decades, hydrogen has been regarded as a potential substitute for fossil fuels, based on the concept of so-called hydrogen economy [4]. It can be produced by photocatalysis from pure water [5], but in the presence of an organic compound, the H_2_ yield can be improved [6]. This process was termed photoreforming [7], by analogy with the thermal steam-reforming [8,9]. The reaction is usually carried out with suspended particles in the liquid phase [10,11,12], but gas-phase systems have recently shown several advantages over liquid-based systems, such as easy catalyst and product recovery, reduced light losses by scattering, and no leaching issues [13]. One drawback of this process is the requirement of gaseous or volatile compounds [14,15,16]. 

Ethanol represents a promising volatile feedstock candidate, since it can be potentially produced on a large scale from renewable non-food lignocellulosic biomass [17]. Despite this prospect, ethanol cannot be completely converted to CO_2_ and H_2_, the expected product of the photoreforming reaction; rather, it undergoes photodehydrogenation to acetaldehyde [16,18], which represents a harmful by-product [19] that should be properly disposed of. 

Despite these results hindering the utilization of ethanol as a hydrogen source, the high selectivity toward photodehydrogenation [13,16] can be useful for chemicals production in biorefineries [20], with the advantages of very mild conditions being necessary for photocatalysis [13]. Indeed, acetaldehyde can be converted to different valuable chemicals, such as acetic acid, acetic anhydride, ethyl acetate [21], and butadiene [22,23]. 

Titanium dioxide (TiO_2_) is the most studied photocatalytic material because of its cheapness, availability, safety, chemical and photochemical stability [24]. This material can be synthetized by several techniques [25,26,27,28], as well as using benchmark materials (i.e., Evonik P25) [29,30]. All these synthetic approaches afford to materials with different structural and morphological properties, which in turn affect the photocatalytic activity [31]. The precipitation technique is widely used for thermal catalysis [32,33], but has been less reported in photocatalysis [34,35], although it has some advantages, such as there being no organic solvent or additives involved, and the avoidance of pressurized equipment. In addition to semiconductor design itself, surface functionalization, in particular its modification with co-catalysts [36], plays an important role. These materials display a dual function, acting as both electron-sink, thus reducing their recombination with holes within the semiconductor [37], and as a true active site for hydrogen evolution [38]. Several co-catalysts have been proposed, such as noble metals [29,30], transition metals [39], and their oxides [40] and sulfides [41]; the most used of these are gold [16,18] and platinum [29,42], which are very effective, but expensive. Being cheap, quite abundant and safe, copper has recently drawn attention as a co-catalyst for photoreforming [43], exhibiting activity comparable to that of noble metals [13]. Copper (II) oxide (CuO) is one of the most studied materials [28,40,44], exhibiting a higher stability in air than other copper oxidation states [45]. The CuO co-catalyst is usually introduced by impregnation [46], but Yoong et al. proposed another simple synthetic approach, named complex-assisted precipitation (CP), using glycerol as ligand to assist the precipitation, and allowing to a highly dispersed CuO material with improved yield compared to the impregnated sample [47]. Furthermore, Chen et al. suggested that a highly dispersed CuO monolayer with higher reactivity can be obtained at low loadings by the CP method [48]. Such improved reactivity was ascribed to the rising of conduction band (CB) energy of nano-size CuO, thus improving its reduction capability [49]. Nonetheless, CuO was observed to be unstable under the irradiation, converting to Cu_2_O, claimed to be the true active site for hydrogen evolution [50]. Therefore, the reason for improved reactivity on narrow-size CuO cannot be related to its CB.

Through this work, the gas phase photodehydrogenation of ethanol was studied by using CuO/TiO_2_ nanocomposites as photocatalysts. In particular, CuO was introduced in both benchmark P25 and precipitated TiO_2_ (lab-made) through incipient wetness impregnation and CP methods. The latter technique was modified by using two different organic oxygenated ligands alternatively to the previously reported glycerol. The nanocomposites were finally tested and characterized to understand the effect of different co-catalyst introduction techniques on different TiO_2_ materials. 

## 2. Materials and Methods 

### 2.1. Materials

The following reagents were used as received: TiOSO_4_∙xH_2_O∙yH_2_SO_4_ (Ti assay >29%, Sigma Aldrich, Milan, Italy), sodium hydroxide (assay >97%, Carlo Erba, Milan, Italy), Cu(NO_3_)_2_∙3H_2_O (assay >99%, Sigma Aldrich), 1,3-propanediol (assay 98%, Sigma Aldrich), citric acid (assay >99%, Sigma Aldrich) and 2-propanol (assay 99.8%, Sigma Aldrich). A benchmark TiO_2_ (P25) was purchased from Evonik (Essen, Germany).

### 2.2. Synthesis of Photocatalysts

The titania and copper-promoted titania samples examined in this study are listed and labelled in Table 1.

#### 2.2.1. Titanium Dioxide Synthesis 

The precipitated TiO_2_ was prepared through a method previously reported by our research group [51]. Briefly, a titanyl sulphate (TiOSO_4_) solution was added dropwise to a beaker containing deionized water under vigorous stirring, keeping pH of 7 by adding a sodium hydroxide (NaOH) solution. The obtained suspension was aged at 60 °C (20 h), filtered and washed with deionized water to remove dissolved ions (sodium and sulphates). The wet titanium hydroxide paste was air-dried at 110 °C (18 h) and finally air annealed at 400 °C (4 h). The resulting material was labelled as TiO_2_. 

#### 2.2.2. Copper Oxide Loading by Impregnation

Copper oxide was introduced on both benchmark P25 and lab-made TiO_2_ by incipient wetness impregnation [52]. Cu(NO_3_)_2_∙3H_2_O precursor was dissolved in water and then added dropwise to the titania powder. Two different copper loadings were chosen: 0.5% wt. and 1.0% wt. After drying (110 °C, 18 h), the materials were air annealed at 400 °C (1 h) and the nanocomposites were obtained. The P25-based nanocomposites were labelled as I0.5Cu/P25 and I1.0Cu/P25 (0.5 and 1.0 wt. % Cu, respectively). The TiO_2_-based material was labelled as I1.0Cu/TiO_2_ (1.0 wt. % loading). 

#### 2.2.3. Copper Oxide Loading by Complex-Precipitation 

Copper oxide was also introduced on both P25 and TiO_2_ by complex-assisted precipitation [47]. This approach is usually performed with glycerol as ligand [48]. In the present work, two different ligands were chosen: 1,3-propanediol and citric acid. The titanium dioxide powder was dispersed in 200 mL of deionized water, then an aqueous solution of Cu(NO_3_)_2_∙3H_2_O and the selected ligand was added. A ligand-Cu molar ratio of 3 was chosen with 1,3-propanediol, while 2 was chosen with citric acid. As for the impregnated samples, 0.5 wt. % and 1.0 wt. %Cu loadings were chosen. The suspension was stirred vigorously for 30 minutes. Then a 0.5 M NaOH solution was added until reaching pH 12, and kept under stirring for 1 h. After filtering and washing, the powder was dried at 110 °C (18 h) and finally air annealed at 400 °C (1 h). The P25-based materials were labelled as D0.5Cu/P25 and D1.0Cu/P25 (0.5 and 1.0 wt. % Cu loading, respectively) if 1,3-propanediol was used, whereas they were labelled as C0.5Cu/P25 and C1.0Cu/P25 (0.5 and 1.0 wt. % Cu loading, respectively) if citric acid was employed. D1.0Cu/TiO_2_ nanocomposite was prepared using TiO_2_, in which 1.0% wt. Cu was introduced with 1,3-propanediol as ligand during the synthesis. 

### 2.3. Characterization of the Nanocomposite Materials

X-ray Diffraction (XRD) patterns were collected on a Bruker D8 Advance powder diffractometer (Billerica, MA, USA) with a sealed X-ray tube (copper anode; operating conditions, 40 kV and 40 mA and a Si(Li) solid state detector (Sol-X) set to discriminate the Cu Kα radiation. Apertures of divergence, receiving, and detector slits were 2.0 mm, 2.0 mm, and 0.2 mm, respectively. Data scans were performed in the 2θ range 5–75° with 0.02° step size and counting times of 3 s/step. Quantitative phase analysis and crystallite size determination were performed using the Rietveld method as implemented in the TOPAS v.4 program (Bruker AXS, Billerica, MA, USA) using the fundamental parameters approach for line-profile fitting. The determination of the crystallite size was accomplished by the Double-Voigt approach and calculated as volume-weighted mean column heights based on integral breadths of peaks.

N_2_ adsorption–desorption isotherms at −196° C were performed using a Micromeritics ASAP 2000 analyzer (Norcross, GA, USA). All samples were previously outgassed at 200 °C (2 h). The mesopore volume was measured as the adsorbed amount of N_2_ after capillary condensation. The surface area was evaluated using the standard BET equation [53]. Single-point surface area values were calculated from the standard BET equation considering only the adsorbed-desorbed gas amount at P/P^0^ = 0.3.

Thermal analyses (TG-DTA) were performed on a NETZSCH STA 409 PC/PG Instrument (Selb, Germany) in air flux (20 mL∙min^‒1^) using a 10 °C∙min^−1^ temperature ramp in the 20–800 °C temperature range. 

The actual amount of Cu in the nanocomposites was determined, after dissolution of the samples in boiling aqua regia, by flame atomic adsorption spectroscopy (FAAS) using a PerkinElmer Analyst 100 (Waltham, MA, USA).

Temperature programmed reduction (TPR) experiments were carried out on a lab-made equipment; each sample (50 mg) was heated at 10 °C∙min^−1^ from 20 to 1000 °C in a 5% H_2_/Ar mixtures (40 mL∙min^−1^ at standard temperature and pressure, STP). The outlet gases were analyzed by a Gow-Mac TCD (Bethlehem, PA, USA).

High-resolution Transmission electron microscopy (HRTEM) measurements were performed with a JEOL 3010-UHR instrument (Tokyo, Japan) operating at 300 kV and equipped with a LaB_6_ filament. Digital micrographs were acquired by a Gatan (2k × 2k)-pixel Ultrascan1000 CCD camera (Pleasanton, CA, USA) and processed by Gatan digital micrograph (Pleasanton, CA, USA). Before the measurements, to obtain a good dispersion of the sample particles and to avoid any modification induced by the use of a solvent, the powders were briefly contacted with the Cu grids coated with lacey carbon, resulting in the adhesion of some particles to the TEM grid by electrostatic interactions. 

Diffuse reflectance (DR) UV–Vis-NIR spectra were collected at r.t. on a Varian Cary 5000 spectrophotometer (Palo Alto, CA, USA) with an integrating sphere attachment using BaSO_4_ powder as an internal reference, working in the 50,000–4000 cm^−1^ range. UV–Vis-NIR spectra of the as prepared samples were reported in the Kubelka-Munk function [f (R_∞_) = (1 − R_∞_)^2^/2R_∞_; R_∞_ = reflectance of an “infinitely thick” layer of the sample [54]. 

The FTIR spectra were recorded in transmission mode at a resolution of 2.0 cm^−1^ on a Perkin-Elmer 2000 spectrometer (equipped with a MCT detector) (Waltham, MA, USA), with the samples in self-supporting pellets introduced in a cell allowing thermal treatments in controlled atmospheres and spectrum scanning at controlled temperatures (from −170 °C to room temperature, r.t.). From each spectrum, the spectrum collected before the inlet of the CO probe (or of ethanol) was used as background. All reported spectra were background subtracted and normalized to the weight of the pellets.

To monitor by CO adsorption the nature of the exposed active sites and the effect of the interaction with ethanol, the photocatalysts were simply outgassed at r.t. below 10^−3^ mbar for 10 min. The samples were then cooled to −170 °C before the inlet of 5 mbar CO. After CO desorption and heating up to r.t. the sample was contacted with 5 mbar ethanol and spectra were collected at decreasing ethanol pressures. Then a second CO inlet at −170 °C was performed.

X-ray photoelectron spectroscopy (XPS) analysis of samples I, D, anc C was performed by a Perkin-Elmer Φ 5600ci spectrometer (Eden Prairie, MN, USA), with non-monochromatic Al Kα source (1486.6 eV). The pressure was in the 10^–6^ Pa range. The surface of the analyzed region was smaller than 1 mm^2^. Wide range survey spectra were recorded for all the sample. Single spectra were recorded for Ti2p, Cu2p, O1s, and C1s regions. The binding energy (BE) scale calibration was checked by the position of both Au4f_7/2_ and Cu2p_3/2_ bands in pure metal samples, falling at 84.0 eV and 932.6 eV, respectively [55]. All the BE values are referred to the Fermi level. The raw XPS spectra were fitted using a non-linear least-square fitting program adopting a Shirley-type background and Gaussian–Lorentzian peak shapes for all the peaks (XPSPEAK41 free software, 4.1, Raymund W.M. Kwok, Hong Kong, China). The BE correction from the surface charging evidenced during analysis (around 2 eV) was done by using an internal reference (Ti2p_3/2_ band centered at 458.8 eV in TiO_2_ compound) [55]. The BE values uncertainty was not larger than 0.2 eV. The atomic composition of the analyzed region (about 10 nm of thickness from the surface) was estimated by the area under the different XPS curves obtained by the fitting procedure, using sensitivity factors provided by Θ V5.4A software (Eden Prairie, MN, USA), with a final uncertainty of the atomic composition of the different elements lower than 10%.

UV-Vis spectra of liquid samples were collected at r.t. on a Perkin Elmer Lambda 2 spectrophotometer (Überlingen, Germany), using 1 cm thick optical glass cuvette and working in the 400–800 nm range with a 120 nm/min scan rate.

### 2.4. Photocatalytic Activity Tests

Gas-phase ethanol photodehydrogenation tests were carried out in a borate glass thin film reactor (33 mm × 18 mm × 2 mm) described in the previous work [51]. A suspension of the catalyst powder (1 mg) in 2-propanol was added to the light-exposed side of the reactor, letting the solvent to evaporate. The prepared reactor was dried in air at 110 °C (1 h) to completely remove the solvent. The reaction mixture was generated by bubbling He (1.5 mL∙min^‒1^) through a 35% v/v ethanol solution (1:6 ethanol-water molar ratio) in deionized water kept at 40 °C: a gaseous mixture containing 11 ± 1% v/v of ethanol and 3.9 ± 0.8 water-ethanol molar ratio was obtained. After reaching a stable composition of the gaseous mixture, the reactor was opened and the process was carried out under a continuous flow of reactants (dynamic conditions) for 3 h. The photocatalyst was irradiated using a 125 W mercury UVA lamp (Helios Italquartz s.r.l., Cambiago, Italy) with emission range of 315–400 nm equipped with a tubular quartz shield to select the 365 nm emission wavelength and an average irradiance of 50 W∙m^−2^, controlled with a Delta Ohm HD 2302.0 photo-radiometer (Caselle di Selvazzano, Italy) and a LP 471 probe (PSE, Steinfurt, Germany).

The reaction mixture was analyzed by a gas chromatographer (HP 5890, Hewlett-Packard Company, Palo Alto, CA, USA) equipped with two columns, Porapak Q and molecular sieve, and a TCD detector. Quantitative analysis of the gaseous stream was performed through calibration curves of ethanol, acetaldehyde, water and hydrogen. Activity results were expressed in ethanol conversion (X_EtOH_) [56], as expressed in Equation (1):

(1) $${X\;}_{\mathrm{EtOH}}=\;\frac{C{\left(0\right)}_{\mathrm{EtOH}}-C{\left(t\right)}_{\mathrm{EtOH}}}{C{\left(0\right)}_{\mathrm{EtOH}}},$$

where C(0)_EtOH_ is the inlet ethanol concentration, and C(t)_EtOH_ is the outlet concentration at time t. When hydrogen was detected, the activity was also expressed as turnover frequency (TOF):

(2) $$\mathrm{TOF}\;=\frac{F_{H_{2}}\left(\mathrm{mol}\cdot s^{-1}\right)}{m_{cat}\left(g\right)},$$

where F_H2_ is the molar flow of hydrogen and m_cat_ is the mass of used photocatalyst.

The photon-efficiency was determined by the apparent quantum yield (Φ), according to IUPAC recommendation [57], as reported in Equations (2) and (3):

(3) $$\Phi =\;\frac{{\mathrm{required}\;e}^{-}\cdot H_{2}\left(\mathrm{mol}\right)}{\mathrm{incident}\;\mathrm{photons}\left(\mathrm{mol}\right)}\times 100,$$

(4) $$\Phi =\;\frac{2\cdot F_{\mathrm{mol}}\left(\mathrm{mol}\cdot s^{-1}\right)\cdot H_{2}\left(\%\right)\cdot h\left(J\cdot s\right)\cdot c\left(m\cdot s^{-1}\right)\cdot N_{A}}{I\left(W\cdot m^{-2}\right)\cdot A\left(m^{2}\right)\cdot \lambda \left(m\right)},$$

where 2 is the number of electrons required for H^+^ reduction to H_2_, F_mol_ is the total molar flow of inlet gas, H_2_ is the hydrogen concentration (% in volume), h is the Planck’s constant, c the light speed in vacuum, N_A_ the Avogadro number, I the light intensity, A the irradiated area and λ the main emitted wavelength. 

## 3. Results and Discussion

### 3.1. Pristine Titanium Dioxide Materials

The gas-phase reaction of pristine materials, P25 and TiO_2_, yielded only acetaldehyde as detected reaction product, suggesting a good selectivity toward photodehydrogenation (Equation (4)), as has already been reported in other works [16,18]. No hydrogen was detected, probably due to the low sensitivity for hydrogen (below 1%) of the detection system.

CH_3_CH_2_OH → CH_3_CHO + H_2_, (5)

**Figure 1:**
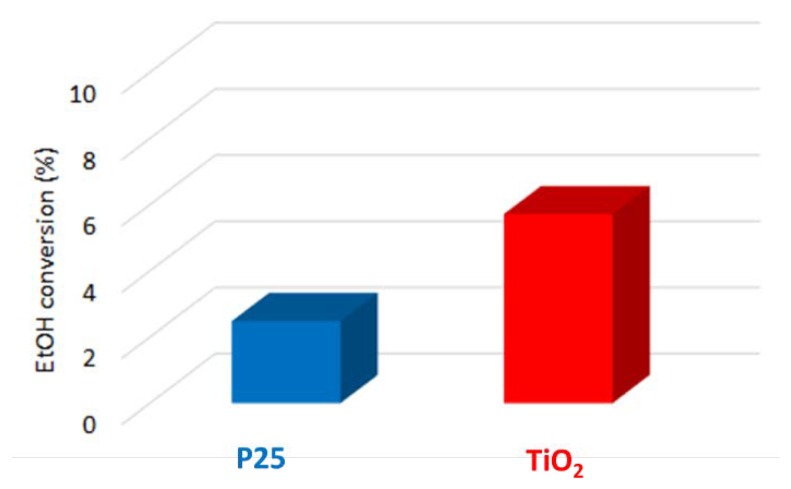
Ethanol conversion of P25 and TiO_2_.

The most interesting result is the improved activity of TiO_2_ compared to P25, as reported in Figure 1.

**Figure 2:**
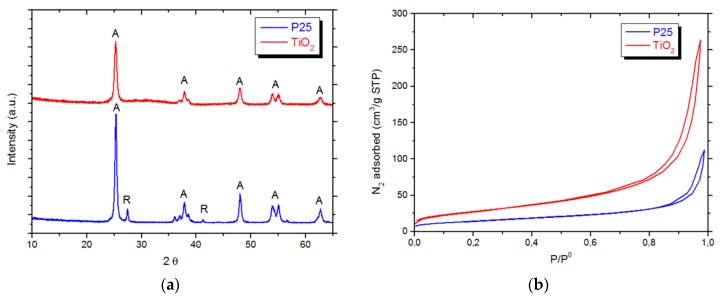
(**a**) XRD spectra of TiO_2_ and P25. Anatase peak are labelled with A and rutile peak with R; (**b**) Nitrogen physisorption isotherms of TiO_2_ and P25.

To establish structure-activity relationships, the physico-chemical properties of the assessed materials were investigated. According to XRD analyses (Figure 2a), P25 is a mixture of two phases: anatase (89%) and rutile (11%), while TiO_2_ is made up of phase-pure anatase. The crystallite size determined by Rietveld analysis for anatase in P25 is larger (18 nm) compared to the lab-made anatase (14 nm). The physisorption isotherms (Figure 2b) reveal that both samples are macro-mesoporous materials, and TiO_2_ exhibits a higher surface area (101 m^2^/g) than P25 (50 m^2^/g) in agreement with XRD findings. A good balance between crystallinity and surface area is known to improve catalytic activity [58]. The enhanced activity of our TiO_2_ can be mainly attributed to the higher surface area, thus probably allowing an enhanced adsorption and oxidation of ethanol. 

### 3.2. Copper Oxide-Titania Nanocomposites

**Figure 3:**
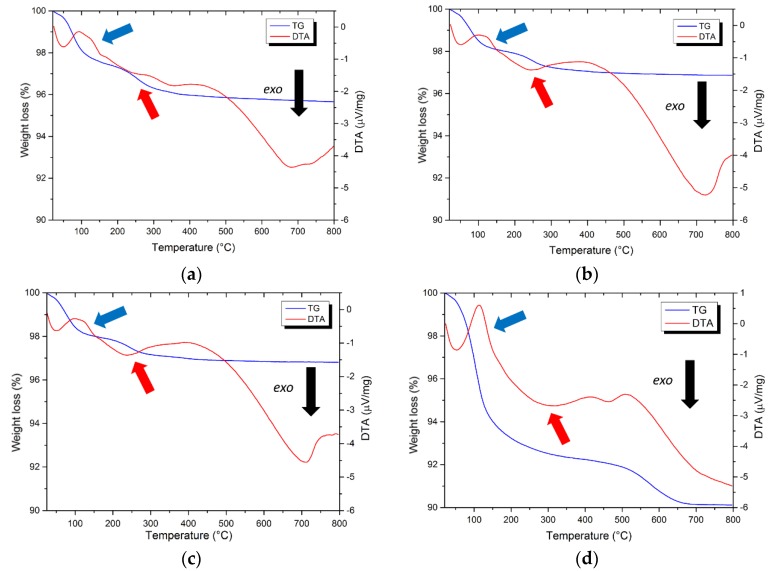
(**a**) TG-DTA of I1.0Cu/P25; (**b**) TG-DTA of D1.0Cu/P25; (**c**) TG-DTA of C1.0Cu/P25 and (**d**) TG-DTA of I1.0Cu/TiO_2._

Copper (II) oxide was used as surface co-catalyst on titania, yielding nanocomposite materials. To efficiently remove residual nitrates and organic compounds, TG-DTA analyses were performed to select the suitable air annealing temperature. All samples exhibited an endothermic peak at 150 °C (red arrows in Figure 3a–d), corresponding to a weight loss, ascribable to a dehydration phenomenon [51]. Exothermic peaks between 200 °C and 400 °C (blue arrows in the Figure 3a–c), corresponding to another weight loss can be related to the decomposition of nitrates [35] on I1.0Cu/P25 and the oxidation of residual organic carbon [59] in the case of D1.0Cu/P25 and C1.0Cu/P25. For I1.0Cu/TiO_2_, the pattern is more complex: a weight loss occurring above 500 °C might be due to residual sulfate decomposition [60]. Finally, all samples exhibited an exothermic peak at 700–800 °C related to anatase to rutile phase transition [61]. Therefore, air annealing at 400 °C guarantees the complete removal of organics and nitrate for all samples while avoiding unwanted phase transition.

#### 3.2.1. Copper Oxide-P25 Nanocomposites

**Figure 4:**
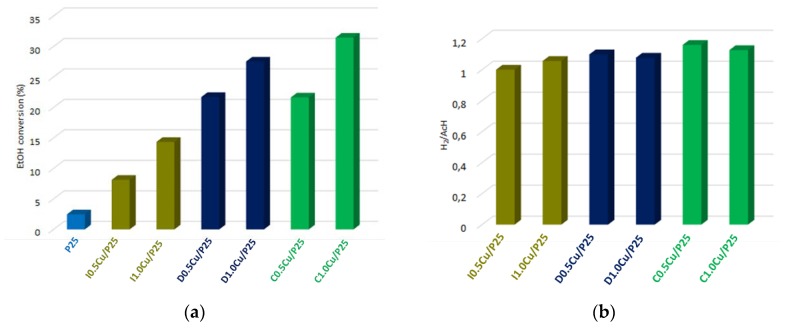
(**a**) Ethanol conversion on P25 and CuO-loaded P25 photocatalysts; (**b**) H_2_/AcH ratio on CuO-loaded P25 photocatalysts.

The addition of CuO co-catalyst to P25 remarkably increased the activity, as reported in Figure 4a, while maintaining the selectivity unchanged, since acetaldehyde was the only detected organic co-product. Moreover, three other observations can be drawn: (i) the higher loading (1.0 wt%) favored higher ethanol conversion regardless to the co-catalyst introduction technique; (ii) the CP-method afforded a more active photocatalyst than the wetness impregnation; and (iii) 1,3-propanediol and citric acid gave similar reactivity of the nanocomposites. Along with acetaldehyde (AcH), hydrogen was also detected and, as reported in Figure 4b, the H_2_/AcH ratios for all samples approached to the unity, suggesting a selective conversion of ethanol to acetaldehyde and confirming the high selectivity of this photodehydrogenation.

The apparent quantum yield (AQY) and turn-over frequency (TOF) can be useful as metrics for assessing the photon utilization efficiency and the productivity of the photocatalytic equipment rig, respectively. Both values confirm the trend observed in ethanol conversion on the CuO-loaded P25 materials. Despite literature reported on photocatalytic equipment working on very different conditions, the assessed AQY and TOF (Table 2) are comparable to those have already reported for gas-phase ethanol photodehydrogenation [16].

To explain the difference in reactivity, flame atomic adsorption spectroscopy (FAAS) and single-point nitrogen physisorption analyses were first performed. As reported in Table 3, no remarkable differences in term of both metal loading and specific surface area (SSA) justify the observed diversities for the nanocomposite materials. The differences among the SSAs reported in Table 3 and that reported in Section 3.1, are due to the different calculation method, i.e., single point measurements and standard BET equation, respectively.

**Figure 5:**
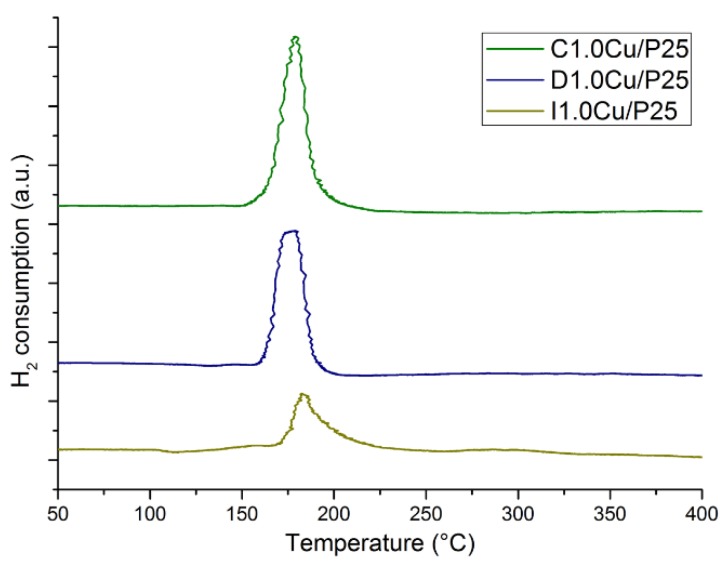
TPR of I1.0Cu/P25, D1.0Cu/P25 and C1.0Cu/P25.

Temperature programmed reduction (TPR) analyses were performed on the samples with 1 wt% Cu loading (Figure 5). All samples exhibited only one reduction peak at 200 °C, suggesting the copper was present in all samples as Cu(II) oxide [62] More interestingly, it was found that the most active photocatalysts (D1.0Cu/P25 and C1.0Cu/P25) exhibited a stronger and sharper reduction peak compared to the impregnated one, namely I1.0Cu/P25. This suggests that CP method affords to more homogeneous and reducible Cu-species, easier and quick convertible to the true active phase, reported to be Cu_2_O [50]. 

**Figure 6:**
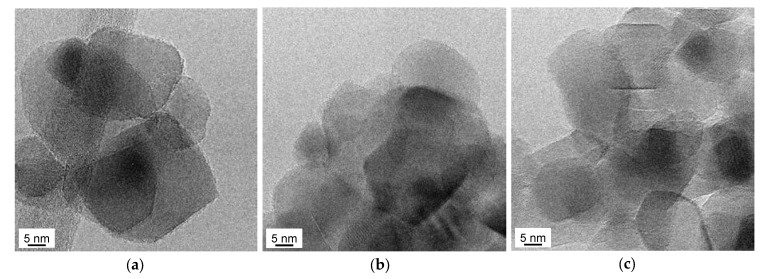
HRTEM images of (**a**) P25; (**b**) D1.0Cu/P25, and (**c**) I1.0Cu/P25. Instrumental magnification 300,000×.

The HRTEM analyses (Figure 6) did not provide evidence for significant differences for either morphology or structure among the D1.0Cu/P25 (6b) and I1.0Cu/P25 (6c) samples and P25 (6a). In particular, the 101 plane was mainly observed in all cases (JCPDS file number) 00-001-0562.

**Figure 7:**
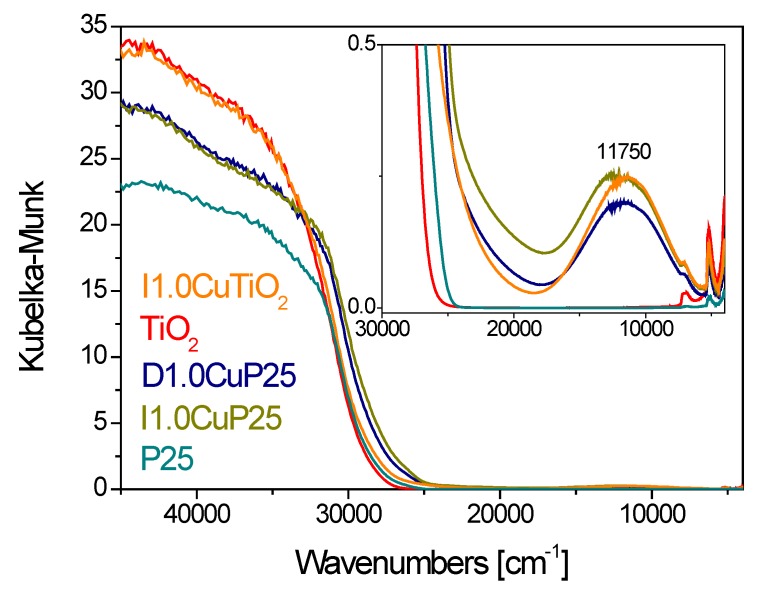
DR UV-Vis-NIR spectra collected on the photocatalysts. Inset: zoom on the absorption band at 11300 cm^−1^. The spectra related to bare P25 and TiO_2_ are also reported for the sake of comparison.

The results of DR UV–Vis-NIR characterization of all samples are reported in Figure 7. The weak absorption band observed at about 11,750 cm^−1^ in Cu-loaded materials is assigned to the d-d transition in Cu(II) species [63,64]. Some differences in position and intensity of this band can be observed among the samples, reasonably related to the selected titania (P25 or TiO_2_) and the CuO introduction method.

The band observed for the CP sample (D1.0Cu/P25, blue curve) has the lowest intensity and is shifted to 11500 cm^−1^, indicating that different copper species, likely to be characterized by a lower energy transition, are formed by CP method. 

Moreover, the Cu-promotion also affects the titania bandgap value for both TiO_2_ and, in particular, P25. This can suggest that the preparation method can also affect the bandgap value.

A comparison between the FTIR spectra collected on I1.0Cu/P25 and D1.0Cu/P25 in the whole spectroscopic range (data not shown) reveals a change in the absorption at frequencies <2500 cm^−1^. This can be ascribed to the erosion of an electronic absorption associated with the presence of free electrons in the P25 conduction band, as a consequence of the population of new energetic levels created when copper is introduced in agreement with the DR UV-Vis-NIR analysis.

**Figure 8:**
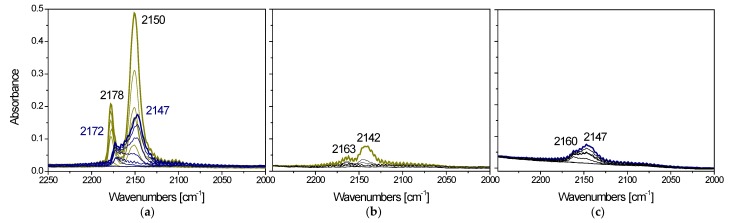
(**a**) Comparison among the FTIR spectra collected upon 5 mbar CO adsorption and at decreasing CO pressures at −170 °C on I1.0Cu/P25 (dark yellow curves) and D1.0Cu/P25 (blue curves); (**b**) spectra of adsorbed CO and decreasing CO pressures at −170 °C collected on I1.0Cu/P25 after ethanol adsorption (dark yellow curve) and further outgassing at r.t. (black curves); (**c**) spectra of adsorbed CO and decreasing CO pressures at −170 °C collected on D1.0Cu/P25 after ethanol adsorption (blue curve) and further outgassing at r.t. (black curves).

Subsequent CO adsorption experiments were performed at low temperature to monitor the surface exposed sites including the hydroxyl groups of titania. Figure 8a shows the comparison among the spectra collected upon 5 mbar CO adsorption and at reducing CO pressures at −173 °C on I1.0Cu/P25 and D1.0Cu/P25. 

Neither Cu^+^ nor Cu^0^ were observed [65] on either I1.0Cu/P25 and D1.0Cu/P25, confirming, as also suggested by TPR findings, that only Cu^2+^ was present on all samples.

Both samples exhibit a band at 2147 (2150) cm^−1^ (Figure 8a) ascribable to CO interacting with titania hydroxyl groups [66], more intense on I1.0Cu/P25 rather than on D1.0Cu/P25, thus suggesting a more hydroxyl-rich surface for the former. Moreover, I1.0Cu/P25 reveals a band at 2172 (2178) cm^−1^ (Figure 8a) assigned to Ti^4+^-CO species [65], showing residual Ti^4+^ surface sites on this material. By contrast, D1.0Cu/P25 did not show any bands related to Ti^4+^-CO, while a component at 2160 cm^−1^ can be assigned to Cu^2+^-CO species [65]. All absorption bands progressively decreased in intensity when the CO pressure was diminished. 

From these results, it can be suggested that the CP method leads to a higher coverage of titania surface by CuO, evidenced by the appearance of Cu^2+^-CO band and a decrease in intensity of the band regarded to CO interacting with titania hydroxyl moiety.

**Figure 9:**
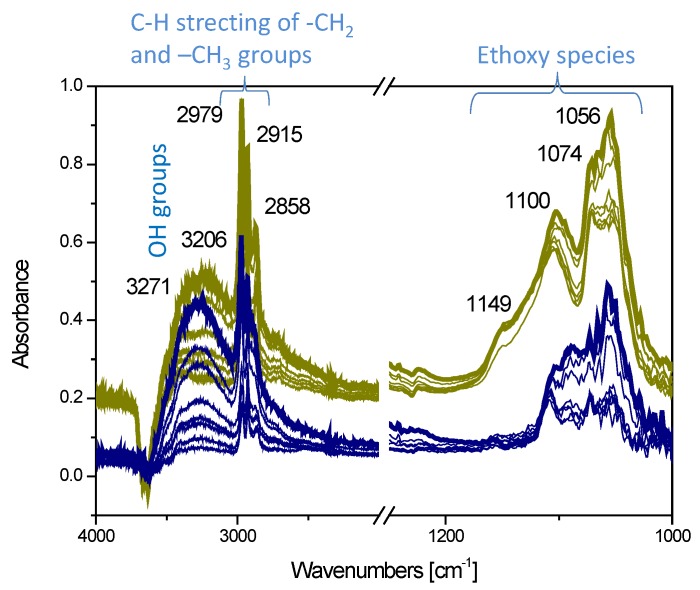
Comparison among the FTIR spectra collected upon 5 mbar ethanol adsorption and at decreasing CO pressures at r.t. on I1.0Cu/P25 (dark yellow curves) and D1.0Cu/P25 (blue curves).

The FTIR spectra collected after ethanol adsorption (5 mbar) at room temperature on the I1.0Cu/P25 and D1.0Cu/P25 photocatalysts simply outgassed at r.t. are shown in Figure 9.

Ethanol was chemisorbed on titania as ethoxy species, onto Ti^4+^ sites, by losing a proton: upon ethanol adsorption at r.t., bands observed at 1100 cm^−1^ (C–C) and 1074 cm^−1^ (C–O) [67] confirmed this kind of chemisorption on both I1.0Cu/P25 and D1.0Cu/P25 (Figure 9). Between the two samples, I1.0Cu/P25 exhibited the most intense bands and revealed a further component at 1149 cm^-1^, suggesting a stronger and different adsorption mode of ethanol on this material. At higher frequencies, peaks in the 2800–3000 cm^−1^ region were attributed to the combination of ν(CH_3_) and ν(CH_2_) vibrational modes of ethanol, and the broad absorption at 3200 cm^−1^ was due to the alcohol O–H stretching mode [68].

The CO adsorption experiments performed at low temperature after having dosed ethanol on the two samples (Figure 8b,c) revealed that ethanol and CO competed particularly for the same Ti^4+^ sites, as shown by the decreased intensity of the carboxylic bands. This is particularly evident for I1.0Cu/P25, whereas free Cu^2+^ sites are observed at the surface of D1.0Cu/P25. 

From these results it can be proposed that the lower photoactivity of I1.0Cu/P25 compared to the CP-based samples (D1.0Cu/P25 and C1.0Cu/P25) can be also ascribed to the stronger adsorption of ethanol, likely to occur particularly onto Ti^4+^ sites, which in turn hinder the photoconversion of the reactant.

XPS survey spectra suggest that only elements C, Ti, O and Cu are present at the samples surface over the detection limit. In all samples carbon atoms are present (see Table 4), originating a C1s band centered in the 248.7–248.9 eV binding energy range (after charging correction): this BE value range is usually related to carbon contamination by the environment. The Cu amounts detected by XPS is almost doubled with respect to those obtained from FAAS analysis (mol %), meaning that the metal oxide, introduced from the surface, remains mainly located into the first few nm. 

The Ti2p band shows the typical shape for TiO_2_ in all samples, with the two components (due to spin-orbit splitting) at the correct energy distance of 5.5 eV. The O1s band is centered at 529.9–530.1 eV (depending on the sample), with the typical BE value range shown by oxygen in titanium dioxide compounds [55]. 

XPS also evidenced the presence of bands related to copper, an element characterized by limited binding energy shift among its different oxidation states. Cu^0^ and Cu^+^ actually originate very similar Cu2p signals, whose 2p_3/2_ component lay in between 932.0 and 932.7 eV of BE [55]. The analysis of the CuLMM band [69] can more precisely discriminate these oxidation states but, being very weak, in our case, it was not possible to achieve useful information.

**Figure 10:**
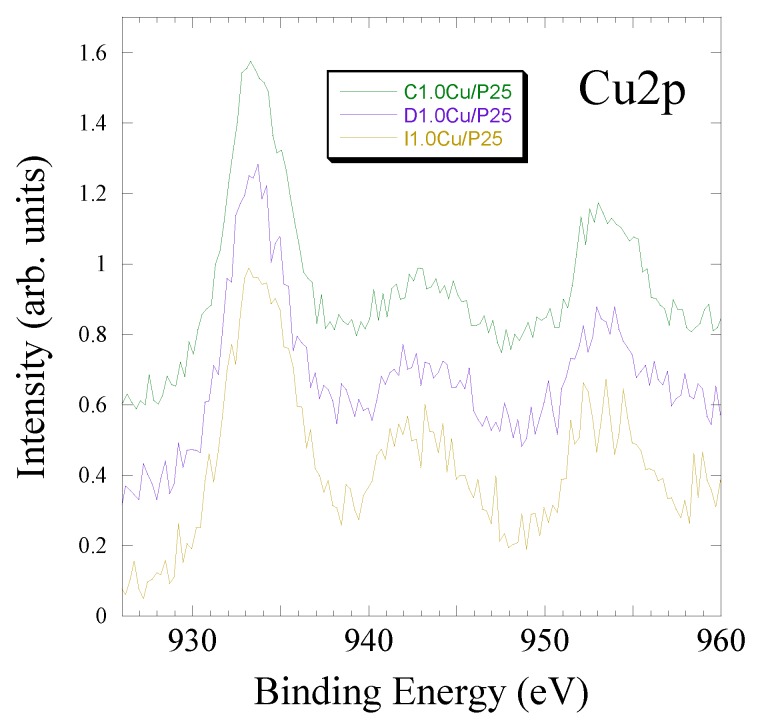
XPS Cu2p bands recorded on the three samples (starting from the bottom, samples I1.0Cu/P25, D1.0Cu/P25 and C1.0Cu/P25). Binding energy scale was corrected by the surface charging.

Concerning Cu^2+^, the 2p_3/2_ signal exhibits a slightly larger BE value, falling in the range 933.0–933.8 eV [55] Cu^2+^ shows two signals due to spin-orbit splitting (Cu2p_3/2_ and Cu2p_1/2_, at a distance of 20 eV). Moreover, the “shake-up satellite” band can be observed at around 943 eV due to multielectron effects during the X-ray-induced electron emission. This is a kind of "fingerprint" of the Cu^2+^ presence at the analyzed surface. As shown in Figure 10, the Cu2p_3/2_ band is centered around 933.5 eV for all the three samples and, most importantly, the shake-up satellite is clearly visible thus confirming the presence of CuO. 

However, Cu^2+^ is very sensible to X-ray irradiation, showing a marked reduction during the spectra acquisition [70] and a decreasing of the shake-up satellite, as we also observed during analyses. Spectra reported in Figure 10 were recorded immediately after the X-ray source lighting to prevent or limit the reduction of Cu^2+^ during analysis. Considering the simulation of the experimental spectra, it was possible to evidence that at the surface of sample I1.0Cu/P25 the Cu2p band is quite completely originated by copper in +2 oxidation state; in samples C1.0Cu/P25 and D1.0Cu/P25 much probably a small amount of Cu^+^ and/or Cu^0^ is also present at the sample surface (a fraction around 20% of the total copper detected). Comparing these results with TPR (Figure 5) and FTIR (Figure 8a), it can be unambiguously claimed that these Cu^+^/Cu^0^ species are formed during X-ray irradiation. This confirms the hypothesis made from TPR analyses: the easier reducibility of the CuO allows a higher catalytic activity by converting Cu^2+^ into the true active phase. 

CuO loading on P25 lead to a great improvement (up to twelve times) of photohydrogenation activity. The introduction technique of CuO was shown to play a crucial role on the activity enhancement, particularly the CP method afforded CuO species easier to be reduced, and with a better coverage of titania surface. This better dispersion allows an improvement of the boundaries between the co-catalyst, where the reduction occurs, and the titania surface, where the organic molecule is oxidized, thus boosting up the reaction rate [71]. Moreover, the better coverage is also supposed to passivate adsorption sites, likely to be Ti^4+^, where ethanol is likely to strongly interact with the surface as ethoxy species, hampering the dehydrogenation process. 

#### 3.2.2. Copper Oxide-TiO_2_ Nanocomposite

Due to its having the best observed performances of bare lab-made TiO_2_ compared to P25, as shown in Section 3.1, the CuO co-catalyst was introduced onto the former by either impregnation (I1.0Cu/TiO_2_) and CP method (D1.0Cu/TiO_2_). As observed in Section 3.2.1., 1.0 wt% was chosen as metal loading in both method due to the better performances, while 1,3-propanediol was selected as a model ligand for the CP approach since no remarkable differences was seen by using either this molecule or citric acid.

**Figure 11:**
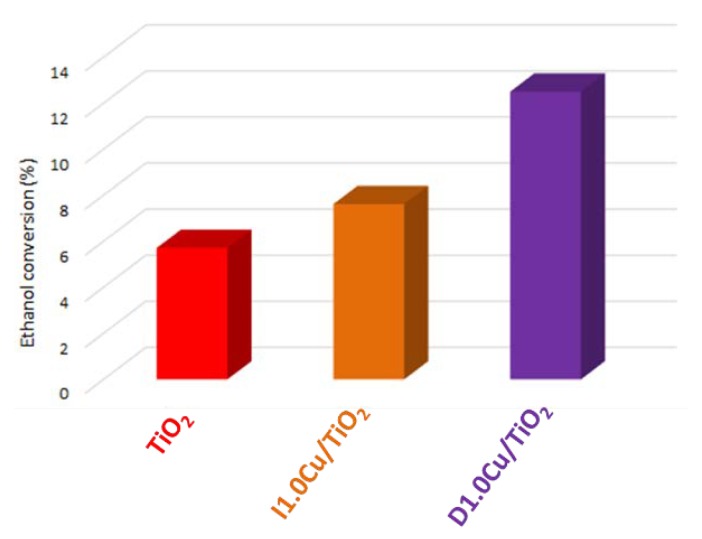
Ethanol conversion on TiO_2_ and CuO-loaded TiO_2_ photocatalysts.

Lab-made TiO_2_-based nanocomposites gave a conversion higher than pristine titania, and the CP method (D1.0Cu/TiO_2_) performed better than wetness impregnation (I1.0Cu/TiO_2_), as reported in Figure 11. Nevertheless, the performances were lower than those expected from the results presented in Section 3.1. The best performing CuO-TiO_2_ nanocomposite (D1.0Cu/TiO_2_) afforded a halved conversion compared to the equivalent P25-based sample (D1.0Cu/P25).

To understand this low conversion, FAAS and single-point nitrogen physisorption analyses were performed (Table 5). All samples exhibited a Cu loading close to the theoretical, and an SSA higher than those measured for P25-based samples. 

**Figure 12:**
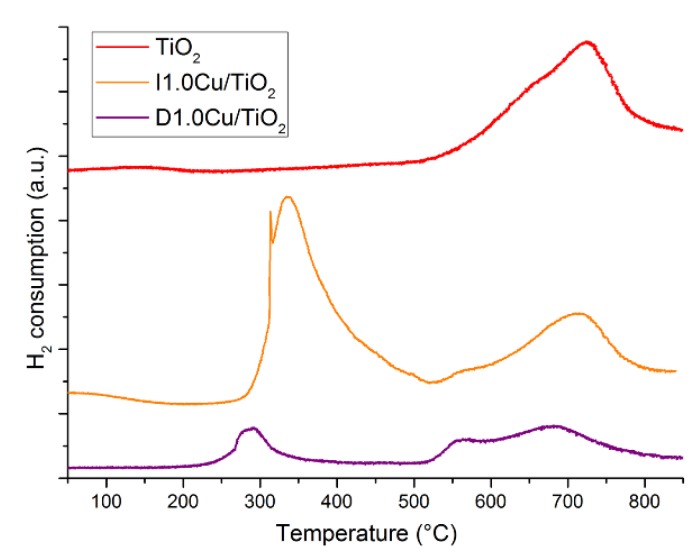
TPR of TiO_2_, I1.0Cu/TiO_2_ and D1.0Cu/TiO_2_.

For a better understanding of such low conversion, TPR analyses were then performed. As reported in Figure 12, TiO_2_-based nanocomposites showed two reduction peaks at 300–400 °C and at 700 °C. The latter can be ascribed to the reduction of titania itself, since a reduction peak was also observed at 700 °C in pristine TiO_2_ (red curve). The former can be related due to the reduction of Cu-species strongly interacting with titania and the less reducibility of these species could lead to the observed limited conversion. It is worth noting that P25 did not exhibit any reduction peak (not shown here). This suggests that lab-made TiO_2_ is somehow more reactive than P25, allowing a stronger interaction with surface Cu-species.

The results of DR UV–Vis-NIR characterization (Figure 7) evidence that the titania band gap of I1.0Cu/TiO_2_ did almost not change upon copper addition, suggesting that the impregnation method did not give a rise to significant electronic modification of TiO_2_. This was further confirmed by contrasting the FTIR spectra of I1.0Cu/TiO_2_ and TiO_2_ in the whole spectroscopic range before CO adsorption (not shown): no electronic effects were observed.

**Figure 13:**
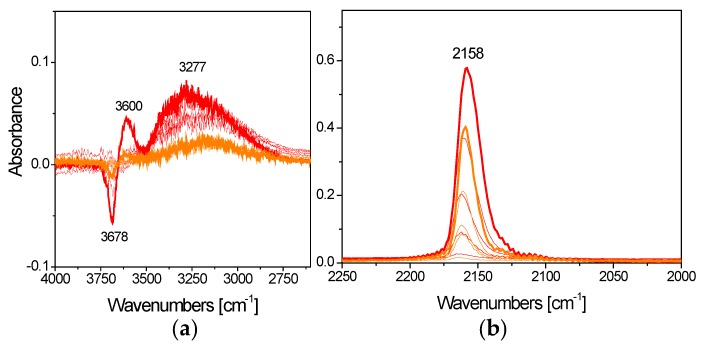
(**a**) Comparison among the FTIR spectra collected upon 5 mbar CO adsorption and at reducing CO pressures at −170 °C on TiO_2_ (red curves) and I1.0Cu/TiO_2_ (orange curves) in the OH stretching; (**b**) and in the carbonylic regions.

The FTIR spectra collected upon 5 mbar CO adsorption and at reducing CO pressures at −170 °C on I1.0Cu/TiO_2_ (orange curves) and TiO_2_ (red curves) are shown in Figure 13.

As for the TiO**_2_** sample, an intense band at 2158 cm^−1^ was observed in the carbonylic region (Figure 13b), due to the interaction of CO with the hydroxyl groups of titania [66]. This assignment is further confirmed by looking at the OH stretching region in Figure 13a. 

These findings indicate the presence of less hydroxyl groups on I1.0Cu/TiO_2_, suggesting that CuO is covering the photocatalyst surface. Indeed, a contribution due to Cu^2+^-CO (2160–2120 cm^−1^) [65], to the band at 2158 cm^−1^ (Figure 13b) cannot be totally excluded. Moreover, no Ti^4+^ sites were detected because a sample outgassing at r.t. and the higher reactivity of lab-made TiO2, resulting in the formation of more surface hydroxyl groups than on P25 (Figure 13b). 

**Figure 14:**
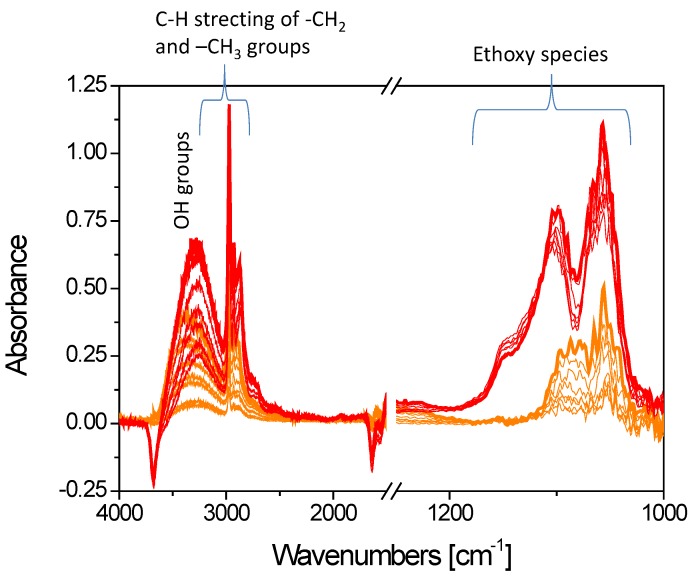
Comparison among the spectra collected upon 5 mbar ethanol adsorption and at reducing CO pressures at r.t. on TiO_2_ (red curves) and I1.0Cu/TiO_2_ (orange curves).

The FTIR spectra collected after ethanol adsorption (5 mbar) at r.t. on the I1.0Cu/TiO**_2_** and TiO**_2_** samples simply outgassed at r.t. are shown in Figure 14. The bands observed in the case of TiO**_2_** are stronger than those observed in I1.0Cu/TiO**_2_**, indicating a more efficient ethanol adsorption on bare TiO_2_. Further CO adsorption experiments performed at low temperature on these samples (not shown) confirmed that ethanol and CO competed for the same sites, as already observed for the P25-containing photocatalysts.

As observed for P25, the presence of Cu species seems to mitigate the strong ethanol adsorption, likely by covering the sites where the reactant is strongly adsorbed. Contrary to P25, in this case since no Ti^4+^ was detected by CO adsorption at low temperature, the sites involved in the interaction with ethanol are reasonably the hydroxyl groups of titania.

Despite the effect of the copper loading which was an increase in ethanol conversion on TiO_2_, in particular when the CP method was employed (Figure 11), the improvement was lower than that achieved with the P25 copper-loaded samples (Figure 4a). The reason of this limited improvement can be ascribed to a lower reducibility of the Cu-species, that strongly interact with the lab-made titanium dioxide due to its higher intrinsic reactivity with respect to P25.

#### 3.2.3. Mechanism of Complex-Precipitation

**Figure 15:**
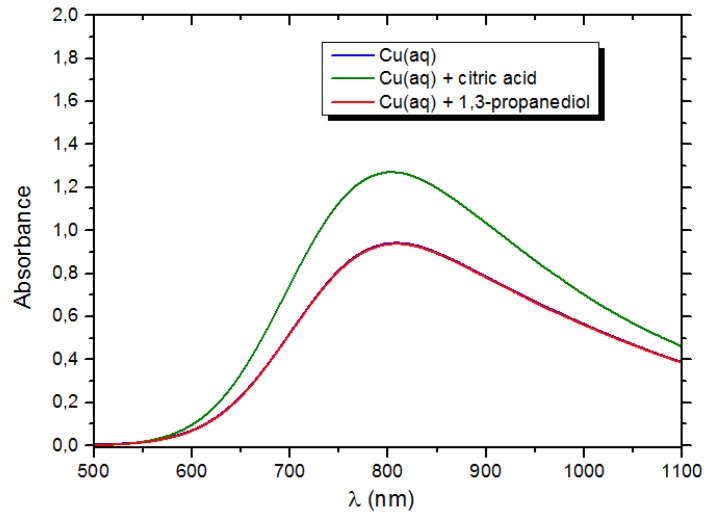
Uv-Vis spectrum of aqueous copper solutions, with and without organic ligands. 1:3 and 1:2 metal-to-ligand molar ratios were used for 1,3-propanediol and citric acid, respectively.

Previous works concerning complex-precipitation in photocatalysis have mainly been focused on the characterization of the material, and no mechanism has yet been proposed [47,48]. UV-visible spectroscopy was used to evaluate how the complexation of Cu ions occurs. As reported in Figure 15, despite an increase of absorbance occurred when citric acid is added, no remarkable shifts of the Cu^2+^ adsorption band were observed, and thus no strong electronic interaction is likely to occur. 

**Figure 16:**
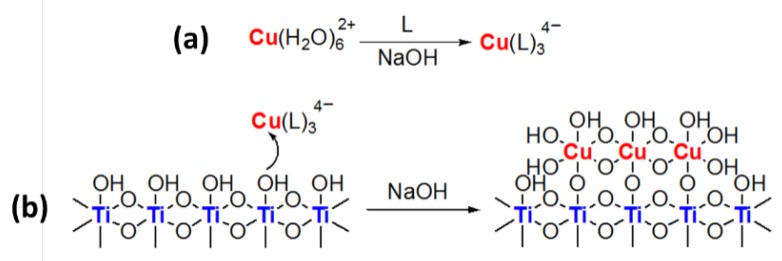
Supposed complex-precipitation mechanism: (**a**) complexation; (**b**) grafting and precipitation.

This finding could suggest a simplified mechanism, as reported in Figure 16. Since the CP method was performed through a gradual pH adjustment by NaOH addition, we propose a two-step mechanism: first, copper ions were coordinated by the ligands, being deprotonated by the alkaline medium (Figure 16a). Then, the formed complex was grafted onto titania surface and, upon increasing the pH, definitely converted to copper (II) hydroxide [Cu(OH)_2_] (Figure 16b). The role of the ligand was to act as a retardant of rapid growth of coarse Cu(OH)_2_ nanoparticles, allowing a higher dispersion of copper. Finally, as evidenced by FTIR (Section 3.2.1.), due to the acidic nature of Ti^4+^ surface sites [66], a direct interaction with hydroxyl ions from the alkaline reaction medium was likely to completely passivate them.

Simultaneous grafting and complexation cannot be excluded, and further studies will be required to fully elucidate the overall mechanism of complex-precipitation technique. 

## 4. Conclusions

We reported a simple synthetic approach for preparing active nanocomposite photocatalysts, based on earth-abundant elements, for the selective photodehydrogenation of ethanol to acetaldehyde. The introduction method of the co-catalyst (CuO) has shown to have a remarkable impact on the activity. In particular, when the complex-precipitation (CP) technique was employed, a highly dispersed and more reducible CuO than the impregnated one was obtained. Moreover, CuO species cover the titania surface, resulting in the passivation of the adsorption sites where ethanol can be adsorbed. These properties were seen to be beneficial for activity improvement. By carefully tuning the synthesis, a 12-fold improvement in ethanol conversion was achieved (on C1.0Cu/P25 sample) compared to pristine benchmark titania (P25), with a 23% quantum efficiency and a productivity of 7.5 mmol∙g^−1^∙h^−1^. Moreover, all nanocomposites exhibited a selectivity toward photodehydrogenation approaching 100%. Concerning the high surface area of lab-made titania samples, despite a two-fold ethanol conversion improvement, it was observed that the introduction of copper led to a modest improvement in activity. This result could be explained by assuming a stronger interaction of copper with the lab-made titania, intrinsically more reactive than P25. Nonetheless, beside the role as co-catalyst, the metal was shown to be beneficial for the photoactivity by decreasing adsorption sites where ethanol can be strongly adsorbed. Finally, a mechanism for CP synthetic technique was proposed: a metal complex was formed in situ, during the precipitation, with the simultaneous or successive grafting to titania surface, ending up with the formation of grafted Cu(OH)_2_. 

Despite the farness from large-scale applications, photocatalysis can be an appealing approach for chemical production in bio-refineries. Activity and stability improvement are still challenging for an industrial application. Nonetheless, the knowledge of the main bottleneck of these processes, as we demonstrated through the present work, plays a crucial role for the development of a catalyst fulfilling large-scale applications.

## Figures and Tables

**Table 1:** Titania and copper-promoted titania materials.

Titania	Copper-Loading (%) and Introduction Technique	Label
Benchmark (P25)	/	P25
Lab-made TiO_2_	/	TiO_2_
Benchmark (P25)	0.5%‒wetness impregnation	I0.5Cu/P25
Benchmark (P25)	1.0%‒wetness impregnation	I1.0Cu/P25
Benchmark (P25)	0.5%‒complex precipitation with 1,3-propanediol	D0.5Cu/P25
Benchmark (P25)	1.0%‒complex precipitation with 1,3-propanediol	D1.0Cu/P25
Benchmark (P25)	0.5%‒complex precipitation with citric acid	C0.5Cu/P25
Benchmark (P25)	1.0%‒complex precipitation with citric acid	C1.0Cu/P25
Lab-made TiO_2_	1.0%‒wetness impregnation	I1.0Cu/TiO_2_
Lab-made TiO_2_	1.0%‒complex precipitation with 1,3-propanediol	D1.0Cu/TiO_2_

**Table 2:** AQY and TOF of CuO-loaded P25 photocatalysts.

Sample	AQY (%)	TOF (mmol∙g^−1^∙h^−1^)
I0.5Cu/P25	13 ± 1	4.4 ± 0.5
I1.0Cu/P25	17 ± 2	5.5 ± 0.6
D0.5Cu/P25	19.0 ± 0.3	6.2 ± 0.1
D1.0Cu/P25	21 ± 1	7.1 ± 0.2
C0.5Cu/P25	21 ± 1	6.8 ± 0.4
C1.0Cu/P25	23 ± 2	7.5 ± 0.6

**Table 3:** Cu actual loading (%) determined by FAAS and specific surface area (S_BET_) by nitrogen physisorption.

Sample	Cu (%)	S_BET_ (m^2^/g)
P25	-	40
I0.5Cu/P25	0.41	41
I1.0Cu/P25	0.43	42
D0.5Cu/P25	0.43	44
D1.0Cu/P25	0.93	42
C0.5Cu/P25	0.99	45
C1.0Cu/P25	0.98	43

**Table 4:** Atomic fraction (%) of Cu, Ti, O, and C as obtained by XPS data. Values are rounded off to keep the correct number of significant figures.

	I1.0Cu/P25	D1.0Cu/P25	C1.0Cu/P25
Cu	2.3	2.4	2.8
Ti	27	23	24
O	63	57	64
C	7	18	9

**Table 5:** Cu actual loading (%) determined by FAAS and specific surface area (S_BET_) by nitrogen physisorption.

Sample	Cu (%)	S_BET_ (m^2^/g)
TiO_2_	-	101
I1.0Cu/TiO_2_	0.89	71
D1.0Cu/TiO_2_	0.94	101
